# Carbohydrate and Fat Oxidation in Muscle Assessed with Exercise Calorimetry in 6465 Subjects

**DOI:** 10.3390/metabo16020121

**Published:** 2026-02-09

**Authors:** Jean-Frédéric Brun, Emmanuel Varlet, Justine Myzia, Emmanuelle Varlet-Marie, Eric Raynaud de Mauverger, Jacques Mercier

**Affiliations:** 1 Unité d’Explorations Métaboliques et Musculaires (UEMM), Département de Physiologie Clinique, Hôpital Lapeyronie-CHU de Montpellier, Université de Montpellier, 34295 Montpellier, France; emmanuel.varlet@etu.umontpellier.fr (E.V.);; 2 UFR des Sciences Pharmaceutiques et Biologiques, Laboratoire du Département de Physicochimie et Biophysique, Institut des Biomolécules Max Mousseron (IBMM), Université de Montpellier, 34093 Montpellier, France; e-varlet_marie@chu-montpellier.fr

**Keywords:** lipid metabolism, skeletal muscle, calorimetry, LIPOXmax, FATmax, training, lipid oxidation, fat, maximal fat oxidation (MFO), fat metabolism, indirect calorimetry, peak fat oxidation, substrate oxidation, carbohydrate oxidation, carbohydrate cost of the watt (CCW)

## Abstract

Background/Objectives: Exercise calorimetry provides a means to quantify the relative contributions of lipid and carbohydrate (CHO) oxidation across a range of exercise intensities. Although lipid oxidation capacity has been widely studied—particularly in relation to exercise prescription for individuals with obesity—the factors governing CHO oxidation during exercise are less clearly defined. This study therefore aimed to investigate, within a large single-center cohort, not only the established determinants of maximal lipid oxidation (LIPOXmax) but also those influencing CHO oxidation. Methods: Exercise calorimetry was performed in a cohort of 6465 individuals (4561 women and 1904 men; mean age 46.5 years; mean BMI 33.6 kg/m^2^). Two principal physiological indices were derived: LIPOXmax, defined as the exercise intensity eliciting maximal rates of fat oxidation, and the carbohydrate cost of the watt (CCW), defined as the slope characterizing the relationship between CHO oxidation and power output. Results: LIPOXmax showed positive associations with lean and muscle mass, and negative associations with fat mass and age, supporting the notion that greater muscle mass enhances the capacity for fat oxidation. Although men demonstrated higher absolute maximal fat oxidation rates, adjustment for body composition revealed that women exhibited relatively higher lipid oxidation (+30%, *p* < 0.001), occurring at a greater percentage of $\dot{V}$O_2max_ (+9.2%, *p* < 0.001). Furthermore, the carbohydrate cost of the watt was significantly elevated in women (+17.8% compared with men). CCW was positively correlated with BMI, fat mass, and age, and negatively correlated with muscle mass, LIPOXmax, and the crossover point—that is, the exercise intensity at which CHO becomes the predominant substrate. Discussion and Conclusions: Individuals with higher adiposity exhibited a greater reliance on carbohydrate oxidation, whereas leaner individuals preferentially oxidized lipids at comparable exercise intensities. These observations reinforce the reciprocal interplay between lipid and carbohydrate metabolism during exercise and highlight the substantial influence of body composition, age, and sex. Notably, this study provides the first comprehensive characterization of the determinants of CHO oxidation during exercise, identifying sex, age, and adiposity as major contributing factors. This underexplored facet of metabolic flexibility may hold practical relevance in clinical contexts such as obesity or susceptibility to exercise-induced hypoglycemia.

## 1. Introduction

The metabolic activity of exercising skeletal muscle can be evaluated indirectly and non-invasively through exercise calorimetry. This method, which relies on the measurement of respiratory gas exchange, enables the quantification of carbohydrate and lipid oxidation rates during physical exertion [1,2,3,4,5]. Exercise calorimetry has contributed to the development of targeted strategies for prescribing physical activity in obesity and metabolic disorders by identifying the exercise intensity that elicits maximal lipid oxidation [6,7,8,9]. Endurance training performed at this intensity has been implemented with encouraging outcomes in the context of obesity management [6,7,8,9].

Accumulating evidence indicates that lipid oxidation rises progressively with increasing exercise intensity, reaches a peak—commonly termed LIPOXmax or FATmax—at approximately 40–50% of maximal aerobic capacity, and subsequently declines, becoming nearly extinguished at higher intensities referred to as LIPOXzero [8] or FATmin [5], typically above 60% of maximal aerobic capacity. In contrast, carbohydrate oxidation continues to increase beyond this threshold. This asymmetric, bell-shaped lipid oxidation profile exhibits substantial interindividual and interpopulation variability and is modulated by numerous factors, including training status, sex, adiposity, dietary patterns, diabetes, hormonal milieu, and pharmacological treatments, as extensively reviewed elsewhere [10].

Exercise calorimetry also enables the quantification of carbohydrate (CHO) oxidation, although this dimension has received considerably less attention. CHO oxidation increases progressively with rising exercise intensity. At low-to-moderate intensities, this increase is approximately linear and can be characterized by the slope of the relationship between CHO oxidation rate and mechanical power output, termed the carbohydrate cost of the watt (CCW) [11,12]. At maximal and supramaximal intensities, the relationship steepens and adopts an exponential profile [13].

Because exercise calorimetry remains insufficiently implemented in routine practice, most published studies are based on relatively small sample sizes [6,7,8,9,10]. At our center, exercise calorimetry has been systematically performed since 2004 to guide individualized exercise prescription in obese and diabetic patients, as well as in athletes and in specific clinical contexts such as anorexia nervosa. This long-standing clinical and research activity has led to the accumulation of an extensive database spanning more than two decades.

The availability of this dataset offered a unique opportunity to delineate the statistical determinants of lipid and carbohydrate oxidation during exercise. Particular emphasis was placed on carbohydrate oxidation, a dimension that remains comparatively underexplored in the existing literature. Accordingly, the primary objective of this study was to identify the determinants of LIPOXmax and the carbohydrate cost of the watt within a large and heterogeneous population. Secondary objectives included comparing these parameters between men and women and assessing their variation across the age spectrum.

## 2. Material and Methods

### 2.1. Subjects

Exercise calorimetry, following the protocol developed in our center and published in 2001, has been routinely implemented since 1997 [3,6] to guide low-intensity endurance training across a range of clinical and athletic contexts, including obesity, diabetes, and various sports disciplines. Beginning in 2004, all tests successfully completed were systematically entered into an Excel database, while tests terminated prematurely were excluded.

As of 2025, the database comprises 6465 individuals (mean age 46.45 ± 0.58 years; range 8–92.4 years), including 4561 women and 1904 men, with broadly comparable age distributions (women: 45.68 ± 0.68 years; men: 48.29 ± 1.11 years) and similar BMI values (women: 33.77 ± 0.50 kg/m^2^; men: 33.11 ± 0.76 kg/m^2^).

Key exclusion criteria included acute illness, uncontrolled cardiovascular disease, inability to complete the test, and body weight exceeding 150 kg, corresponding to the upper limit of our cycle ergometer.

Overall, this database encompasses individuals with diverse clinical profiles—including type 2 diabetes, metabolic syndrome, and athletic populations—and thus represents a heterogeneous clinical cohort.

Characteristics of patients are presented on Table 1.

In the sample, BMI values ranged from 15.1 to 61.5 kg/m^2^. The distribution by category was as follows:

- Underweight (BMI < 18.5): 0.1% of women and 0.2% of men.
- Normal weight (BMI 18.5–25): 7.9% of women and 7% of men.
- Overweight (BMI 25–30): 21.7% of women and 25% of men.
- Obesity class I (BMI 30–35): 30.2% of women and 33.7% of men.
- Obesity class II (BMI 35–40): 23.9% of women and 20.9% of men.
- Obesity class III (BMI > 40): 16.1% of women and 13.2% of men.

Overall, 70.2% of women and 67.8% of men fell within the obesity range, while only 7.9% of women and 7% of men had a BMI in the normal range.

Accordingly, this sample represents a wide range of BMIs but includes a large number of patients suffering from overweight and obesity.

### 2.2. Body Composition

Body composition was evaluated using a BIACORPUS RX 4000 bioelectrical impedance analyzer (MEDI CAL HealthCare GmbH, Greschbachstr. 6a, 76229 Karlsruhe, Germany), which operates with an alternave 50 kHz current [14,15]. The collected impedance data were processed using BodyComp 8.4 software, which estimates segmental fat and fat-free mass through equations developed from the manufacturer’s reference population. Skeletal muscle mass was calculated using the Janssen prediction equation, which relies on hand-to-foot resistance measured at 50 kHz [16]. This equation is as follows:

Muscle mass (kg) = H^2^/R_50_ × 0.401 + gender [M = 1/F = 0] × 3.825 + age (years) × (−0.0731) + 5.102

where H is body height and R_50_ is hand-to-foot resistance measured at 50 kHz.

### 2.3. Exercise Test

Participants performed the exercise assessment in the morning following a 12 h overnight fast. The protocol consisted of five steady-state stages of six minutes each, initially targeted at 20, 30, 40, 50, and 60% of predicted maximal power (Pmax).

Theoretical predicted Pmax values used to target workloads in each subject were calculated according to the very classical empirical formulae of Wasserman [17] that are implemented in our home-made software for calculation of the balance of substrates. First, predicted weight (PW) is calculated from height using sex- and obesity-specific formulas: for men PW = 0.79 × height (cm) − 60.7, for women PW = 0.79 × height (cm) − 68.2, and for obese subjects PW = 0.65 × height (cm) − 42.8, with the actual weight (BW) compared to PW to determine whether an obesity correction must be applied. PW is then used to estimate maximal oxygen uptake (VO_2max_), according to the formula: VO_2max_ (mL/min) = [50.72 × PW (kg) − 20.40 × age (years) + 5.61] × (1 if male/0.85 if female).

Finally, $\dot{V}$O_2max_ is converted to Pmax using a standard mechanical efficiency factor (10 mL O_2_ ≈ 1 watt at maximal effort).

These workloads could be adjusted during the procedure depending on the evolution of the respiratory exchange ratio (RER = $\dot{V}$CO_2_/$\dot{V}$O_2_) to ensure that values were recorded both below and above 0.9, the threshold corresponding to the Crossover Point defined later. Tests were conducted on an electromagnetically braked cycle ergometer (Ergoline Bosch 500, Ergoline GmbH, 72475 Bitz, Germany). A standard 12-lead ECG continuously tracked heart rate and cardiac activity throughout the protocol. Ventilatory and metabolic variables were obtained using a computerized breath-by-breath analysis system (COSMED Quark CPET, COSMED Srl, Pomezia, Italy).

#### Predicted $\dot{V}$VO_2max_ (ACSM Method)

Because a precise measurement of $\dot{V}$VO_2_ peak is not essential for exercise calorimetry, maximal oxygen uptake was estimated after exercise was completed, by extrapolating the individual $\dot{V}$VO_2_–heart-rate relationship to the theoretical age-predicted maximal heart rate, following ACSM recommendations ($\dot{V}$VO_2max__ACSM) [18].

### 2.4. Exercise Calorimetry

During each workload, $\dot{V}$O_2_ and $\dot{V}$CO_2_ (mL/min) were recorded to compute the non-protein RER. Rates of lipid oxidation (Lipox) and carbohydrate oxidation (Glucox) were obtained from gas-exchange data using the classical non-protein respiratory quotient method previously described [7,8]. This approach provides substrate-utilization values at each exercise intensity. After curve smoothing, two indices describing the shift from lipid toward carbohydrate metabolism were extracted: the maximal lipid-oxidation point (LIPOXmax) and the Crossover Point (COP).

The crossover point has been defined by the team of George Brooks [13] as the point where CHO represents more than 70% of energy used for muscle activity. It can be easily calculated from the equations of calorimetry presented below that this corresponds to a RER value of ≈0.9.

LIPOXmax corresponds to the workload at which lipid oxidation reaches its peak before declining as carbohydrate use continues to rise.

Following earlier work on long-duration steady-state calorimetry [19,20], Perez-Martin et al. proposed a protocol [21] comprising five submaximal stages of 6 min each—the duration required to obtain stable gas-exchange values. The test is carried out on a cycle ergometer with continuous $\dot{V}$O_2_/$\dot{V}$CO_2_ monitoring and ECG surveillance. Workloads are initially set to ~30, 40, 50, and 60% of predicted maximal power but can be adapted according to the RER in order to obtain values below and above 0.9 (the level crossover point), and at least one stage above RER = 1, where fat oxidation essentially drops to zero (LIPOXzero or “FATmin”). Gas-exchange measurements from minutes 5–6 of each stage, assumed to reflect steady state, are used to compute substrate oxidation through standard indirect-calorimetry equations [22,23,24]:

(1) $$\mathrm{Carbohydrate}\;\mathrm{oxidation}\;(\mathrm{mg}/\min)\;=\;4.585\;\dot{V}{\mathrm{CO}}_{2}\;-\;3.2255\;\dot{V}O_{2}$$

(2) $$\mathrm{Lipid}\;\mathrm{oxidation}\;(\mathrm{mg}/\min)\;=\;-1.7012\;\dot{V}{\mathrm{CO}}_{2}\;+\;1.6946\;\dot{V}O_{2}$$

The rationale for using 6 min steps and performing calculations on the values of the 5–6th min is based on a study by McRae and coworkers [22] which indicates that, at this time, the CO_2_ production from bicarbonate buffers becomes negligible. There has been controversy about the best duration of the steps, and many authors prefer to use shorter steps [23].

Nevertheless, these various protocols provide quite the same information. They show that the increase in lipid oxidation displays an asymmetrical dome-shaped curve. This curve culminates at the level of MFO at an intensity which is termed in this protocol the LIPOXmax, and then lipid oxidation decreases at higher power intensities. The power intensity where it becomes equal to zero is the point where RER is equal to 1 and is termed the LIPOXzero (or FATmin).

The empirical formula (Equation (2)) that gives the lipid oxidation rate is, as reminded above:


(3) $$\mathrm{Lipid}\;\mathrm{oxidation}\;(\mathrm{mg}/\min)\;=\;-1.7\;\dot{V}{\mathrm{CO}}_{2}\;+\;1.7\;\dot{V}O_{2}$$

It is easy to deduce from this formula that the relation between power (P) and oxidation of lipids (Lox) displays an asymmetrical dome-shaped curve of the form:

Lox = A.P (1 − RER)(4)

Derivation of this curve enables us to calculate the power intensity at which lipid oxidation becomes maximal, which is the point where the derivative becomes equal to zero. Therefore, the LIPOXmax calculation is only an application of the equation of lipid oxidation used in calorimetry and is model-independent [8].

The reproducibility of the LIPOXmax has been investigated in several studies and it was found to range between 5.02% and 11.4%, and on average 8.7% [8].

### 2.5. Measuring the Kinetics of Carbohydrate Oxidation During Exercise

**Figure 1:**
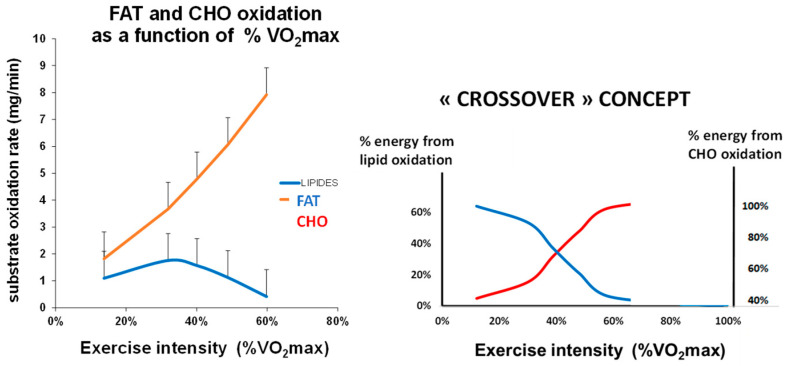
(**left**) Average curve of fat and CHO oxidation as function of exercise intensity in the whole sample of 6465 individuals. It can be seen that over the range of intensities applied during the test CHO oxidation can be approximately modelized as a straight line (carbohydrate cost of the watt) whose slope is on the average 0.24 mg/min/kg/watt. (**right**) Reconstruction from the data of this study of the classical Brook’s picture of the “crossover concept” [13].

Although the increase in carbohydrate oxidation above resting levels follows an exponential pattern [13], within the range of intensities typically used during graded exercise calorimetry, it behaves, in practice, as an approximately linear function of power output (see Figure 1). The slope of this relationship—representing the carbohydrate cost required to generate one watt of mechanical power—is referred to as the carbohydrate cost of the watt (CCW) [11,12]. As detailed in Aloulou et al. [11], comparative analyses of various mathematical models demonstrate that a linear function provides an adequate and robust description of the rise in CHO oxidation with increasing power output. Alternative curvilinear models do not yield higher correlation coefficients on average, and variance comparisons reveal no significant improvement in data dispersion; indeed, the linear model exhibits the lowest variance among those tested. Although curvilinear models may also produce high correlation coefficients, the linear formulation remains the simplest and most consistently reliable representation of the relationship between CHO oxidation and power output during exercise. In that study, the mean carbohydrate cost per watt (±SEM) was reported as 0.22 ± 0.001 mg min^−1^ kg^−1^ W^−1^, with the upper boundary of the first quintile at 0.16 and the lower boundary of the fifth quintile at 0.29 mg min^−1^ kg^−1^ W^−1^ [11].

Intraindividual variability of this parameter was investigated in that previous study. Previous work has shown that the carbohydrate cost of the watt is reproducible with a mean difference of 9.2 ± 28.4% and a coefficient of variation for paired values of 15.9% [11].

### 2.6. Statistical Analysis

Data are reported as means ± SD in order to show the distribution of the values, since SEMs are close to 10^−9^. Statistical analysis was with the Sigmastat package (Version 3.0, Jandel Scientific, Erkrath, Germany). Given the exploratory nature and the very large sample size, *p*-values were interpreted descriptively, with emphasis on effect sizes rather than formal multiplicity correction. Comparisons were performed with two-ways analysis of variance (ANOVA) or Student *t*-test (after the normality of distribution was assessed) when two samples were compared. We validated the assumptions of normality and homogeneity of variances before performing the ANOVA test and calculating the adjusted *p*-values for multiple comparisons using a Tukey test. To account for multiple testing, *p*-values were adjusted for multiple comparisons using Bonferroni’s correction. Correlations were calculated on Microsoft EXCEL. Differences were considered significant at *p* < 0.05.

## 3. Results

**Figure 2:**
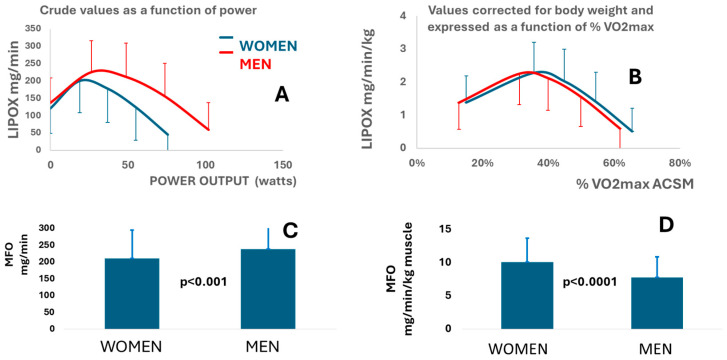
Comparison of men and women for the parameters of lipid oxidation. Error bars represent SD. (**A**) Comparison of fat oxidation expressed as crude values plotted against crude power intensity in men compared to women (ANOVA time effect and group effect *p* < 0.001); (**B**) comparison of fat oxidation corrected for body weight and plotted against % of maximal aerobic capacity (ANOVA time effect and group effect *p* < 0.001); (**C**) comparison of maximal fat oxidation rate expressed as crude values in men compared with women (*p* < 0.001); (**D**) comparison of maximal fat oxidation rate corrected for muscle mass in women compared to men (*p* < 0.001).

This large dataset confirms that, although men exhibit higher absolute maximal fat oxidation rates and reach this peak at higher power outputs, women display slightly higher maximal lipid oxidation rates when values are expressed relative to body weight. Moreover, in women, this peak occurs at a marginally higher exercise intensity (Figure 2).

More precisely, when lipid oxidation is corrected for anthropometry, women exhibit a higher (+30%) maximal ability to oxidize lipids during exercise, expressed by muscle mass (10.10 ± 0.16 vs. 7.78 ± 0.20 *p* < 0.001) (Table 2), and fat oxidation peaks at a slightly higher (+9.2%) percentage of $\dot{V}$O_2max_ ACSM (45.62% ± 0.01 vs. 41.73% ± 0.01 *p* < 0.001) (Table 3).

**Figure 3:**
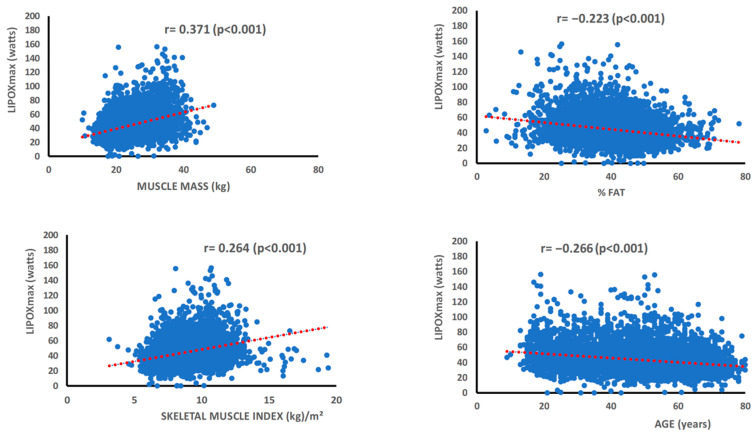
Correlations between parameters quantifying fat oxidation during exercise, age and fatness. **Upper** panel: correlations between the maximal fat oxidation rate at exercise and muscle mass (**left**) and between % fat mass (**right**). **Lower** panel: correlation between LIPOXmax and skeletal muscle index (**left**) and age (**right**).

The principal correlations between LIPOXmax and other physiological parameters are presented in Figure 3. The power output at which maximal lipid oxidation occurs (LIPOXmax) is positively associated with lean body mass (r = 0.325, *p* < 0.001), a relationship largely driven by its muscular component (r = 0.371, *p* < 0.001). LIPOXmax is also positively correlated with the skeletal muscle index (r = 0.264, *p* < 0.001) and with lean mass expressed as a percentage of total body weight (r = 0.197, *p* < 0.001), indicating that it scales with overall muscularity. Conversely, LIPOXmax is negatively associated with fat mass percentage (r = −0.223, *p* < 0.001) and with age (r = −0.266, *p* < 0.001). A strong negative correlation is also observed with the carbohydrate cost per watt (r = −0.544, *p* < 0.001), reflecting the reciprocal balance between carbohydrate and lipid utilization. Finally, maximal fat oxidation (MFO) and LIPOXmax are themselves positively correlated (r = 0.211, *p* < 0.001).

**Figure 4:**
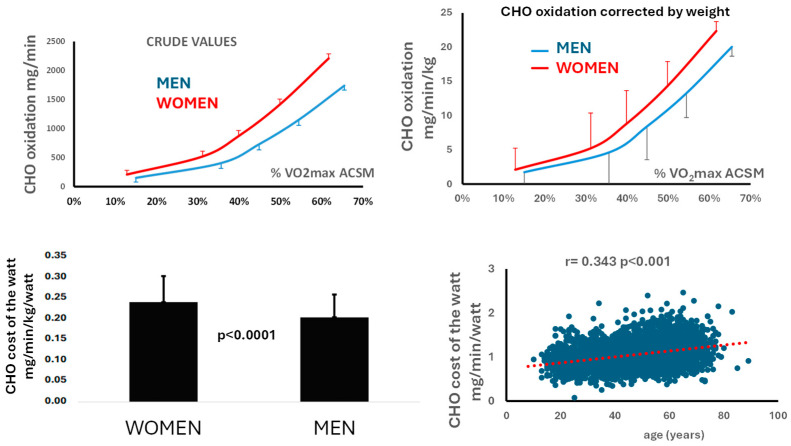
Comparison of men and women for the parameters of Carbohydrate (CHO) oxidation. (**Lower** panel, **right**): carbohydrate cost of the watt is positively correlated with age. (values ± SD).

Carbohydrate oxidation increases progressively with rising power output in both sexes, and within the range of intensities examined, this relationship is approximately linear (Figure 4, upper panel). In women, the rate of increase is 21.48 ± 0.32 mg min^−1^ W^−1^ compared with 20.75 ± 0.48 mg min^−1^ W^−1^ in men (Table 2), resulting in a 17.8% higher carbohydrate cost of the watt in women (0.24 ± 0.004 vs. 0.21 ± 0.005 mg min^−1^ kg^−1^ W^−1^, *p* < 0.001) (Table 2; Figure 4, lower panel, left). The carbohydrate cost of the watt is also positively associated with age (r = 0.343, *p* < 0.001) (Figure 4, lower panel, right).

**Figure 5:**
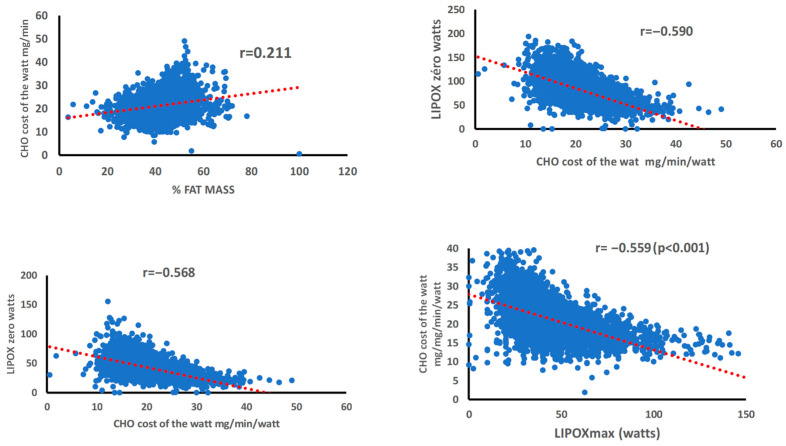
Correlations between parameters quantifying carbohydrate oxidation during exercise, age and fatness. (**Upper** panel, **left**): correlation between the carbohydrate cost of the watt and the percentage of fat mass (r = 0.211 *p* < 0.001); (**Upper** panel, **right**): negative correlation between the carbohydrate cost of the watt and the LIPOX zero, i.e., the intensity level of exercise where CHO becomes the exclusive fuel used for oxidation (r = −0.590 *p* < 0.001). (**Lower** panel, **left**): negative correlation between the carbohydrate cost of the watt and the crossover point expressed as a crude power intensity (r = −0.568 *p* < 0.001). (**Lower** panel, **right**): negative correlation between the carbohydrate cost of the watt corrected for weight and the LIPOXmax expressed as power intensity (r = −0.559 *p* < 0.001).

The carbohydrate cost of the watt expressed as carbohydrate oxidation rate per unit of power (mL/min/watt) is positively correlated with body mass index (r = 0.271 *p* < 0.001) and body fat percentage (r = 0.211 *p* < 0.001), and negatively to the crossover point (r = −0.568 *p* < 0.001) and the LIPOXzero expressed in crude power intensity (r = −0.590 *p* < 0.001) (Figure 5).

When this carbohydrate cost per watt is expressed as carbohydrate oxidation rate per watt and per unit of body weight (mL.min^−1^.kg^−1^.watt^−1^), it is negatively correlated with lean body mass (r = −0.506 *p* < 0.001), muscle mass (r = −0.397 *p* < 0.001), MFO (r = −0.264 *p* < 0.001), the crossover point expressed in crude power (r = −0.546 *p* < 0.001), and LIPOXzero (r = −0.563 *p* < 0.001). The most significant of these correlations are shown in Figure 5.

Stepwise multiple regression analyses were performed to identify the determinants of the substrate-balance parameters described above. Table 4 presents the model for LIPOXmax expressed in absolute power (watts). The model accounted for 15.4% of the variance in the dependent variable (adjusted R^2^ = 0.154, *p* < 0.0001). All predictors retained in the final model were statistically significant (*p* < 0.05), with age exerting the strongest influence (β = −0.260), followed by BMI; both variables were negatively associated with LIPOXmax.

Table 5 presents the regression model for LIPOXmax expressed as a percentage of $\dot{V}$O_2max_. The model accounted for 5.5% of the variance in the dependent variable (adjusted R^2^ = 0.055, *p* < 0.0001). All predictors retained in the final model were statistically significant (*p* < 0.05), with BMI exerting the strongest influence (β = 0.332).

For LIPOXmax expressed either in absolute power or as a percentage of $\dot{V}$O_2max_, Table 4 and Table 5 indicate that the regression models satisfied the assumption of normality but violated homoscedasticity, a limitation that should be taken into account when interpreting the findings. The relatively low adjusted R^2^ values suggest that the variables included in the models are, overall, modest predictors of LIPOXmax, and that additional determinants not assessed in this study may also contribute to its variability.

Table 6 presents the regression model for maximal fat oxidation (MFO). The model accounted for 10.8% of the variance in the dependent variable (adjusted R^2^ = 0.1077, *p* < 0.0001). All predictors retained in the final model were statistically significant (*p* < 0.0001). Muscle mass exerted the strongest influence (β = 0.4809), followed by the skeletal muscle index (SMI; β = −0.3287) and, to a lesser extent, BMI (β = 0.2599).

The model satisfied the assumption of normality but violated homoscedasticity, a limitation that should be taken into account when interpreting these results.

Table 7 presents the regression model for the carbohydrate cost of the watt (CCW). The model accounted for 36.4% of the variance in the dependent variable (adjusted R^2^ = 0.364, *p* < 0.0001). All predictors retained in the final model were statistically significant (*p* < 0.0001). LIPOXmax expressed in watts exerted the strongest influence (β = 0.592), followed by fat-free mass (β = −0.415).

Table 8 shows the regression model for LIPOXzero. This model explained 62.2% of the variance in the dependent variable (adjusted R^2^ = 0.622, *p* < 0.0001). All predictors included were statistically significant (*p* < 0.05). LIPOXmax expressed as a percentage of $\dot{V}$O_2max_ had the strongest effect (β = 0.798), followed by fat mass (kg) (β = −0.166).

For both CCW and LIPOXzero, Table 7 and Table 8 indicate that the models satisfied the assumption of normality but violated homoscedasticity, a limitation that should be taken into account when interpreting these findings.

## 4. Discussion

This large dataset offers a unique opportunity to examine the determinants of substrate utilization during exercise across a broad spectrum of ages and body composition profiles. Our findings first confirm the previously reported influence of sex on lipid oxidation. Although men exhibit higher absolute maximal fat oxidation (MFO) values and reach this peak at higher power outputs, anthropometric adjustment reveals that women possess a greater relative capacity to oxidize lipids during exercise. In women, maximal lipid oxidation also occurs at a significantly higher percentage of maximal aerobic capacity (+9.2%). Moreover, the exercise intensity eliciting maximal lipid oxidation is positively associated with fat-free mass—particularly muscle mass—and negatively associated with adiposity. The two principal indices of lipid metabolism, MFO and LIPOXmax, are strongly correlated with each other and both display inverse associations with indices of carbohydrate (CHO) oxidation. Multivariate analyses further indicate that LIPOXmax is negatively related to age and BMI, whereas MFO is primarily determined by muscle mass and, to a lesser extent, BMI. For CCW, the model identifies LIPOXmax as the strongest predictor, followed by fat-free mass, while for LIPOXzero, LIPOXmax is the dominant determinant, followed by fat mass.

Overall, fat oxidation exhibits modest sex-related differences and shows negative associations with age, fat mass, and adiposity. Its magnitude (MFO) is positively correlated with muscle mass. However, these determinants collectively account for only a limited proportion of the variance in fat oxidation.

A particularly notable finding of our study is the central role of the reciprocal balance between lipid and carbohydrate (CHO) utilization during exercise. Indices of CHO oxidation and those of fat oxidation are strongly and inversely correlated, in line with previous work demonstrating a metabolic interaction between these pathways. This pattern also reinforces the concept that individuals differ in their preferential substrate use: some predominantly oxidize lipids, whereas others—often described as “glucodependent”—rely more heavily on CHO at a given power output [7,8]. Such profiles are observed not only in sedentary, obese, or diabetic individuals [7,8,21], but also in athletes specializing in short, high-intensity efforts [8].

Overall, the observed correlations do not support the existence of a strict proportional relationship between lipid oxidation and any single determinant. Multivariate analyses produce models that account for only a modest proportion of the variance in LIPOXmax and MFO. Consequently, although statistical prediction models may offer approximate estimates [24], exercise calorimetry remains the most reliable approach for determining individualized training intensities for targeted exercise prescription [6,9].

One of the most distinctive contributions of this study lies in the detailed examination of carbohydrate (CHO) oxidation during exercise, a domain that has received comparatively limited attention in the literature. CHO oxidation increases progressively with rising power output in both sexes, as reflected by the carbohydrate cost of the watt (CCW). This increase is 17.8% higher in women than in men and is positively associated with both age and fat mass. The mean CCW values observed in this cohort closely match those previously reported by Aloulou et al. [11], thereby reinforcing earlier findings. CCW is negatively correlated with LIPOXzero—the exercise intensity at which CHO becomes the exclusive substrate—as well as with the crossover point and LIPOXmax. Overall, CHO oxidation tends to rise with advancing age and increasing adiposity, while decreasing with greater fat-free and muscle mass.

Although maximal lipid oxidation was proposed more than 25 years ago as a tool for exercise prescription in obesity and metabolic diseases, it remains insufficiently implemented in practice. Low-intensity exercise designed to maximize lipid oxidation was long perceived as counterintuitive compared with the higher intensities traditionally associated with athletic training. Yet this intensity domain is readily attainable for sedentary or deconditioned individuals, who often tolerate vigorous exercise poorly. Notably, LIPOXmax closely matches the spontaneous walking pace that can be sustained for prolonged periods without dyspnea [25], a form of locomotion that characterized human activity patterns until the 20th century. Prescribing exercise at LIPOXmax therefore represents a return to a physiologically natural mode of movement that has diminished with modern lifestyles. In contrast, low-volume, high-intensity exercise that primarily stimulates CHO oxidation—such as brief running bouts—has frequently been shown to increase appetite and energy intake. While this effect is offset by the substantial energy expenditure of athletes engaging in high training volumes, individuals performing limited amounts of submaximal high-intensity activity may therefore experience paradoxical and unexpected weight gain [26].

Targeted physical activity performed at LIPOXmax has been extensively evaluated and consistently shown to reduce fat mass while preserving lean and muscle mass and improving aerobic fitness, as highlighted in recent meta-analyses [27,28]. Beyond its excellent tolerability and ease of implementation, this training approach demonstrates notable long-term efficacy, supporting sustained weight stabilization for up to eight years following weight loss [29] and enhancing both the magnitude and durability of the weight-reducing effects of bariatric surgery over a five-year period [30,31].

Consistent with previous reports [32,33,34], our findings confirm a sex-related difference in lipid oxidation during exercise. This difference is modest and becomes evident only in large cohorts and after adjustment for anthropometric variables and aerobic capacity [35]. Women exhibit a LIPOXmax occurring at an exercise intensity approximately 9% higher than that observed in men. Although the underlying mechanisms remain incompletely understood, hormonal influences appear to contribute. Studies by Boisseau and colleagues indicate that estradiol enhances lipid oxidation during exercise, whereas progesterone exerts an opposing effect [36,37]. Additional experimental work further supports the role of estrogens in promoting lipolysis and lipid oxidation in both humans and cultured muscle cells [38,39,40].

A central objective of this study was to characterize carbohydrate (CHO) oxidation during exercise. We previously proposed quantifying this parameter using the slope of the relationship between CHO oxidation rate and power output (CCW) [11]. Two additional indices complement this approach: the Brooks and Mercier crossover point [13], which is inversely related to CCW and reflects early CHO predominance (“glucodependence”) [3], and LIPOXzero, which is negatively correlated with both the crossover point and LIPOXmax. Together, these indices support the concept of a tightly regulated balance between lipid and CHO oxidation, consistent with the crossover model. At the molecular level, this balance is explained by mechanisms described by Sahlin [41], whereby increased intramuscular CHO oxidation inhibits fatty acid entry into mitochondria through CPT-1 inhibition mediated by malonyl-CoA and lactate. Accordingly, the rate of CHO oxidation is one of the key factors governing substrate selection during exercise.

CCW was 17.8% higher in women than in men and increased with both age and fat mass, indicating a greater reliance on CHO oxidation in older and obese individuals. In contrast, higher muscle mass was associated with lower CHO oxidation. In our predominantly sedentary and overweight cohort, this pattern likely reflects concurrent declines in muscle mass and metabolic conditioning. Different profiles might be observed in endurance-trained athletes, who were underrepresented in this sample.

We previously demonstrated that CCW typically remains within narrow limits, behaving almost as a biological constant, although it can be markedly elevated in sedentary individuals or in athletes experiencing exercise-induced hypoglycemia [10,11,12]. The present study provides robust reference values, confirming those reported earlier in smaller cohorts [11].

Exercise calorimetry has also been proposed as a tool for assessing metabolic flexibility—the capacity of mitochondria to shift between lipid and carbohydrate (CHO) oxidation [10]. Impaired metabolic flexibility is associated with insulin resistance and type 2 diabetes [42] and can be improved through lifestyle interventions [43,44,45]. The strong inverse relationship observed here between lipid and CHO oxidation supports this concept. In certain situations, however, both lipid oxidation at low intensity and CHO oxidation at high intensity may be simultaneously elevated, as reported in women and in hypothyroid patients treated with L-thyroxine [46]. These observations have contributed to the development of the concept of an optimal fat/CHO oxidation ratio (OLORFOX), which aims to minimize excessive CHO utilization during exercise and has been linked to effects on appetite and weight regulation [47].

Methodologically, substrate balance was assessed using a protocol based on 6 min steady-state exercise stages, originally developed by Perez-Martin et al. [3]. This approach is likely to yield more accurate estimates of substrate oxidation than shorter 3 min stages, which tend to overestimate lipid oxidation and underestimate CHO oxidation in sedentary individuals [48]. Standardization of the test focused on ensuring that measurements captured workloads both above and below a respiratory quotient of 0.9, rather than imposing fixed power outputs, in order to more precisely characterize the substrate crossover zone occurring at this threshold.

This study presents several strengths, most notably its large sample size and the wide range of ages and BMI values represented. However, certain limitations should be acknowledged, including its retrospective, cross-sectional design, the predominance of obese participants, and the lack of information on diet, medication use, and training status. Systematic data on diabetes and dyslipidemia were not available for the entire cohort and therefore could not be incorporated as covariates. Nevertheless, the broad BMI range (15.1–61.5 kg/m^2^) enhances the generalizability and relevance of the findings.

In this study, confidence intervals around correlation coefficients were not reported. Given the very large sample size, these intervals would be extremely narrow, and emphasis was therefore placed on effect sizes instead. Another methodological consideration concerns the well-documented sex differences in substrate oxidation. Our findings confirm that women and men exhibit slightly different metabolic responses during exercise. The absence of stratified or sex-adjusted analyses may somewhat limit the interpretability of these results. However, our data indicate that these sex-related differences are modest and unlikely to substantially affect the overall conclusions. Nonetheless, a more detailed analysis incorporating sex stratification could provide additional insights and may be worthwhile to pursue.

## 5. Conclusions

This large-scale study confirms that lipid oxidation during exercise is influenced by sex, age, and adiposity, and provides the first comprehensive characterization of the determinants of CHO oxidation. It underscores the reciprocal balance between the two principal energy substrates, with a shift toward greater “glucodependence” in older and overweight individuals. These findings support the use of exercise calorimetry to assess metabolic flexibility and to refine exercise prescriptions in the contexts of obesity, insulin resistance, and exercise-related hypoglycemia. Preliminary data from this cohort have already contributed to the development of exercise guidelines aimed at optimizing fat oxidation [28], and the present analysis further reinforces their physiological foundation.

## Figures and Tables

**Table 1:** Anthropometric characteristics of the subjects included in the study (values ± SD). Anthropometry is highly significantly different between males and females in this large sample of unselected subjects but the overall difference in age and BMI is actually very small.

	All Subjects (*n* = 6465)	Females (*n* = 4561)	Males (*n* = 1904)	Comparison *t*-Test
Age (years)	46.45 ± 0.58	45.68 ± 0.68	48.29 ± 1.11	*p* < 0.001
Height (cm)	166.35 ± 2.07	162.73 ± 2.41	175.02 ± 4.01	*p* < 0.001
Weight (kg)	92.93 ± 1.16	89.36 ± 1.32	101.47 ± 2.32	*p* < 0.001
BMI (kg/m^2^) ^a^	33.58 ± 0.42	33.70 ± 0.50	33.11 ± 0.76	*p* < 0.001
Fat free mass (kg)	54.36 ± 0.71	49.62 ± 0.77	66.18 ± 1.62	*p* < 0.001
Muscle mass (kg)	24.30 ± 0.33	21.35 ± 0.34	31.96 ± 0.83	*p* < 0.001
% fat	41.12 ± 0.54	43.91 ± 0.68	34.14 ± 0.83	*p* < 0.001
Waist circumference (cm)	103.29 ± 1.35	100.63 ± 1.56	109.93 ± 2.69	*p* < 0.001
Hip circumference (cm)	113.68 ± 1.49	114.91 ± 1.78	110.59 ± 2.71	*p* < 0.001
$\dot{V}$O_2max_ ACSM ^b^ (mL^−1^.min^−1^.kg^−1^)	25.43 ± 0.32	23.98 ± 0.36	28.91 ± 0.66	*p* < 0.001

^a^ BMI: Body Mass Index. ^b^ $\dot{V}$O_2max_ ACSM: predicted $\dot{V}$O_2max_ by ACSM method.

**Table 2:** Intensity levels of the various flow rates of substrate oxidation during exercise (values ± SD). Anthropometry is highly significantly different between males and females in this large sample of unselected subjects but the overall difference in age and BMI is very small, with average values of the various parameters of balance of substrates during exercise.

	All Subjects (*n* = 6465)	Females (*n* = 4561)	Males (*n* = 1904)	Comparison *t*-Test
MFO ^a^ (mg.min^−1^)	218.66 ± 2.72	210.35 ± 3.11	238.54 ± 5.46	*p* < 0.001
MFO ^a^ corrected for muscle mass (mg.min^−1^.kg^−1^)	9.46 ± 0.13	10.10 ± 0.16	7.78 ± 0.20	*p* < 0.001
MFO ^a^ corrected for fat free mass (mg.min^−1^.kg^−1^)	4.189 ± 1.62	3.63 ± 0.09	4.30 ± 0.07	*p* < 0.001
Crude CCW ^b^ (mg.min^−1^.watt^−1^)	21.27 ± 0.26	21.48 ± 0.32	20.75 ± 0.48	*p* < 0.001
CCW ^b^ corrected for body weight (mg.min^−1^.kg^−1^.watt^−1^)	0.24 ± 0.004	0.25 ± 0.001	0.21 ± 0.005	*p* < 0.001
CCW ^b^ corrected for fat-free mass (mg.min^−1^.kg^−1^.watt^−1^)	0.41 ± 0.01	0.44 ± 0.01	0.32 ± 0.01	*p* < 0.001
CCW ^b^ corrected for muscle mass (mg.min^−1^.kg^−1^.watt^−1^)	0.94 ± 0.01	1.04 ± 0.02	0.67 ± 0.02	*p* < 0.001

^a^ MFO: maximal rate of fat oxidation. ^b^ CCW: Carbohydrate cost of the watt.

**Table 3:** Average values of the various parameters of balance of substrates during exercise (values ± SD).

	All Subjects (*n* = 6465)	Females (*n* = 4561)	Males (*n* = 1904)	Comparison *t*-Test
LIPOX ^a^_(watt)_	44.51 ± 0.55	40.56 ± 0.60	53.94 ± 1.23	*p* < 0.001
LIPOX $\dot{V}$O_2_	990.18 ± 12.31	930.66 ± 13.78	1132.53 ± 25.93	*p* < 0.001
LIPOXmax% $\dot{V}$O_2max_	0.44 ± 0.01	45.62 ± 0.01	41.73 ± 0.01	*p* < 0.001
PCX ^b^_(watt)_	44.50 ± 0.55	40.55 ± 0.60	53.94 ± 1.23	*p* < 0.001
PCX ^b^ $\dot{V}$O_2_	1038.80 ± 12.91	976.72 ± 14.46	1187.30 ± 27.18	*p* < 0.001
LIPOXzero $\dot{V}$O_2_	989.48 ± 12.30	930.54 ± 13.77	1132.55 ± 25.93	*p* < 0.001
LIPOXzero _(watt)_	87.97 ± 1.09	80.06 ± 1.19	106.91 ± 2.45	*p* < 0.001
LIPOXzero% $\dot{V}$O_2max_	0.67 ± 0.01	0.68 ± 0.01	0.65 ± 0.01	*p* < 0.001

^a^ LIPOX: lipid-oxidation point. ^b^ PCX: crossover point.

**Table 4:** Final stepwise multiple regression model for dependent variable LIPOXmax expressed in crude power (watts).

Variable	Coefficient (B)	Standard Error	β	F-to-Remove	*p*
Constant	38.905	2.0995			
Age	−0.265	0.0162	−0.260	267.0	<0.0001
BMI	−0.563	0.0910	−0.253	38.2	<0.0001
FFM	0.330	0.0495	0.159	44.4	<0.0001
Muscle mass	0.486	0.0877	0.125	30.7	<0.0001
Fat mass	0.159	0.0429	0.147	13.7	0.0002

**Table 5:** Final stepwise multiple regression model for dependent variable LIPOXmax expressed as % VO_2max_.

Variable	Coefficient (B)	Standard Error	β	F-to-Remove	*p*
Constant	37.9923	2.4169			
Age	0.0688	0.0144	0.0765	22.79	<0.0001
BMI	0.6516	0.0773	0.3323	71.06	<0.0001
FFM	−0.2440	0.0440	−0.1330	30.79	<0.0001
% fat	−0.1413	0.0604	−0.0786	5.47	0.0194

**Table 6:** Final stepwise multiple regression model for dependent variable MFO expressed as % VO_2max_.

Variable	Coefficient (B)	Standard Error	β	F-to-Remove	*p*
Constant	105.99	11.37			
Age	−0.204	0.0861	−0.0386	5.59	<0.0181
BMI	2.986	0.2630	0.2599	128.94	<0.0001
FFM	−0.711	0.3495	−0.0661	4.14	0.0420
Muscle mass	9.706	1.1295	0.4809	73.84	<0.0001
SMI	−19.563	2.706	−0.3287	52.26	<0.0001

**Table 7:** Final stepwise multiple regression model for dependent variable “Carbohydrate cost of the watt” (CCW).

Variable	Coefficient (B)	Standard Error	β	F-to-Remove	*p*
Constant	4.0488	0.19066			
Age	0.0117	0.00149	0.1096	61.6	<0.0001
FFM	−0.0901	0.00359	−0.4151	631.1	<0.0001
FM	0.0274	0.00169	0.2423	261.1	<0.0001
SMI	0.0724	0.01834	0.0603	15.6	<0.0001
LIPOXmax	0.0618	0.00146	0.5916	1783.1	<0.0001

**Table 8:** Final stepwise multiple regression model for dependent variable “LIPOXzero”.

Variable	Coefficient (B)	Standard Error	β	F-to-Remove	*p*
Constant	0.355075	0.014682			
Age	−0.000373	0.000118	−0.0322	10.05	0.0015
FFM	−0.000886	0.000274	−0.0374	10.48	0.0012
Fat mass	−0.002051	0.000144	−0.1663	203.96	<0.0001
LIPOXmax% $\dot{V}$O_2max_	0.010308	0.000132	0.7984	6089.46	<0.0001

## Data Availability

Due to medical confidentiality and ethical restrictions, the patient data underlying this study are not publicly available and cannot be shared.
